# Prevalence rate of attention deficit hyperactivity disorder (ADHD) and computer vision syndrome (CVS) symptoms predisposition among digital device users of Bangladesh

**DOI:** 10.1186/s43045-022-00176-2

**Published:** 2022-01-25

**Authors:** Z Islam, M Rahman, A H Olive, MK Hasan

**Affiliations:** 1 Department of Pharmacy, East West University, 1212 Aftabnagar Dhaka, Bangladesh; 2 Department of Public Health, North South University, 1229 Dhaka, Bangladesh

**Keywords:** ADHD, CVS, Digital media use, Cognitive inhibition, Correlation

## Abstract

**Background:**

Around 5.29% of the world population is suffering from ADHD, and 60 million people are suffering from CVS, with an increasing rate of prevalence of these disorders. This study aimed to determine the prevalence rate of ADHD and CVS symptoms among the Bangladeshi population.

**Results:**

To assess the aim of the study, a cross-sectional survey was conducted online through stratified sampling, and 197 responses were collected from the participants. Our survey method follows these criteria where the ARSV1.1 standard questionnaire was followed for the ADHD questionnaire, and a self-administered questionnaire was established based on the symptoms of CVS. The male age ranges from 18–24 have the highest value of ADHD (34%) coincided with > 6 h digital device usage (51%), and the Stroop effect is significantly correlated with the ADHD score (0.498, *p* < 0.01). The Stroop effect value is also higher among the males aged 18–24, digital device users for > 6 h (48%).

**Conclusions:**

With the advent of science, it is impossible to avoid digital devices as necessary. Notwithstanding, safe and appropriate use of digital media is a must for healthy living.

## Background

The twenty-first century, known as the age of technology, has exposed people to various technological devices, e.g., television, video games, computers, and cellular technologies, especially people born after 1997 have been exposed to technology [1]. Electronic devices are used more widely in modern times and have become an indispensable part of life. To make life easier, people from all age groups use computers and other technologies more than ever for their professional and personal lives. Nowadays, banking, trading, education, social networking, art & culture largely depend on digital devices [2]. Though the technologies make comfort a certain level, it has an adverse effect on life [3]. The constant technology use can decline brain function and deviation in day-to-day life behavior, as evidenced by the emerging data [4]. The expanding network of technology has drawn the neuroscientists' attention to the burning question of how digital technology may change our brains and behavior. For example, in the positive sense, the older individuals having cognitive disintegration over time could use the technology to help them remain independent longer; however, many seniors with cognitive affliction are unable to adopt new technologies and pass through numerous anomalies [4].

In terms of that, ADHD— a psychiatric condition involving persistent difficulty in sustaining attention; hyperactivity, and impulsivity becomes the most common disease knocking at the door in the triumphant moment of technology progression [5]. A meta-analysis study has reported that around 7% of youth was affected by ADHD from 1981 to 2014, increasing [6]. A recent study revealed that the prevalence rate of ADHD is 5.29% [7]. Due to the COVID-19 outbreak, the prevalence of ADHD has been increased significantly [8]. Moreover, ADHD has been diagnosed as one of the risk factors of COVID-19 [9]. As environmental factors, not related to genetics, are related to ADHD, The advent of digital media and technology stimulates brain dysfunctionality and other hormonal imbalance; it is unknown whether frequent use of digital media may be associated with the symptoms of ADHD [6]. One study also shows that the inattentiveness and cognitive inhibition rises with the Stroop test result for heavy digital device users [10]. Therefore, the concentration and attention deficiency occurred after prolonged use of digital media. Digital media play another detrimental physical role, often leading to a group of symptoms collectively known as CVS (Computer Vision Syndrome) [11]. The American Optometric Association defines CVS as a group of eyes and vision-related problems resulting from excessive and prolonged electronics use. Works that need concentration, such as reading, cause the reduction of the blinking rate of the eye, so the evaporation of tear film is increased and leads to discomfort [12]. Due to computer use, 10 million eye examinations were performed by optometrists per year for visual problems related to computer use [3]. Around 60 million people worldwide suffer from CVS, with 1 million increasing every year [13]. CVS symptoms can range from dry eyes, blurred vision, and eye pain to neck, shoulder, and headaches [3].

According to a report, 47.61 million people of Bangladesh use the internet out of 165.5 million, and the number has increased by around 7.7% from 2020 to 2021 [14]. According to the UN report of 2018, 50% of the Bangladeshi population is aged below 24 years [15]. As the younger generation is more addicted to digital devices, they are more prone to be suffered from ADHD and CVS. Previous studies of ADHD in the Bangladeshi population revealed that it could be liable for adverse consequences [16]. There is no study on the Bangladeshi population for CVS. This study aims to find out the prevalence rate and predisposition of ADHD and CVS among digital device users of Bangladesh.

## Methods

### Participation

The randomly chosen, right-handed, 197 healthy participants (participated in the study by answering all the questions of the study) categorized between *n* = 98 female (50%) and *n* = 99 male (50%), were mainly undergraduate students (77%) from various departments and diversified jobholders (23%) from different areas of Bangladesh. The age range for male and female participants was 18–31 years (male = mean 23.57 ± 3.32 years SD, female = 25.43 ± 3.37 years SD). The participants were mainly frequent computer and other digital device users and had been using these devices for about 5–10 years (44%), > 10 years (31%), 2–4 years (21%), and < 2 years (4%). The inclusion criteria of the participants were:

- Having a digital device.
- Anyone over the age of 18.
- Both sexes.

The exclusion of the participants was based on different factors like:

- Arthritis.
- Previous injuries.
- Color blindness [17].
- Not having a computer or laptop and internet connection.

### Materials and procedures

The data was collected from September 2020 to October 2020 by following the cross-sectional and stratified random sampling methods. In the sampling process, data variables were arranged based on gender, occupation and age-wise. The data collection method followed the structure without missing respondents; all the participants had to respond to the entire incorporated questionnaire. The questionnaire was collected from the World Health Organization Adult ADHD Self-Report Scale (ASRS) constructed in conjunction with the revision of the WHO Composite International Diagnostic Interview (CIDI) for the ADHD symptoms prevalence data compilation [18]. In terms of questionnaires` standardization, it has consistency on sensitivity (68.7%), specificity (99.5%), total classification accuracy (97.9%), and Cohen kappa value (0.76) regarding the adult's symptom acceptability [19]. In our included questions, (1–4) sequence questions represent the severity of the inattention; (5–6) specify the impulsivity and the 7th question represents hyperactivity according to the standard ASRS-v1.1 manual [20]. The ADHD scoring method was followed by the established procedure of ASRS-v1.1, where the process was to mark each question based on the selected option from "Sometimes" to "Very often" [19].

In the scoring method, ADHD scores less than 4 indicate the little disposition of ADHD symptoms, whereas a score 4 or higher than 4 indicates the high prevalence of ADHD in the participants and needs to go for the further diagnostic procedure ASRS-V1.1 symptoms checklist tool [20]. For CVS, the self-administered research-based questions were drawn up, where the questions included demographic information of age, gender, duration of computer use, use of glasses, or contact lenses. At last, ADHD scoring was correlated with the Stroop test effect [21]. The Stroop effect represents the cognitive inhibition of the participants, and then the relationship with the digital media use was correlated. The open-source website PsyToolkit was used to engage the Stroop test, which only runs on the real keyboard. Here, the RT (Reaction Time) was measured comparing incongruent and congruent stimuli along with " + " and "right/wrong" appearing on the screen. In the color word strop task, the following colors, for example: red, yellow, blue, and green, were presented on a black background. The presentation of stimuli within each block was randomized and repeated 15 times per color for 70 trials. The whole Stroop task process lasted approximately 3.5 min. The survey taker solved any confusion regarding the instruction given on the Stroop task or in the questionnaire. Most importantly, their confidentiality and anonymity were maintained. The participants' full consent was taken in the form before participating in the game-task survey analysis.

### Statistical analysis

Data were analyzed using the Statistical Package for Social Sciences software version 20.0 and the Microsoft Excel 2016 for professionals. The correlation between ADHD and CVS is performed by bivariate correlation and linear regression procedure. The bivariate correlation is done by Spearman—Pearson correlation coefficient at the 2-tail significance test. Correlation is significant at the 0.01 level (2-tailed). For CVS, the correlation and significance of data with gender have been estimated by the logistic regression analysis procedure with 95% confidence interval and p-value less than 0.05 have been considered statistically significant.

## Results

Table 1 represents the socio-demographic characteristics of the sample that have been described, along with the percentage and sample number for different variables. First of all, the ratio between female and male participants was ≈50%, and their age ratio was close enough as for 18–24, the range was 52%, and for the age range 25–31, it was 48%. Along with, the hour of computer and any digital device use in the sample was 1–3 h (30%), 4–6 h (28%), and > 6 h (30%). The non-user of the contact lenses and power glass was the majority (60%), and half of the sample had no prior eye problem (≈49%), and the other half had vision problems (24%), dry eye (6%), and (21%) others.

**Table 1:** Socio-demographic characteristics and percentage of the sample

Socio-demographic Characteristics	Sample number (N)	Percentage
Total	197	100%
Gender		
Male	99	50%
Female	98	50%
Age group (in year)		
18–24	102	52%
25–31	95	48%
Occupation		
Student	151	77%
Job holder	46	23%
Spending hours on your digital device?		
1–3 h	59	30%
4–6 h	55	28%
> 6 h	83	30%
Use of Contact lenses /power glasses		
Yes	78	40%
No	119	60%
Using a digital device For years		
< 2 years	8	4%
2–4 years	41	21%
5–10 years	87	44%
> 10 years	61	31%
ADHD score of the population		
Less than 4	119	60%
Equal or Greater than 4	78	40%

**Fig. 1:**
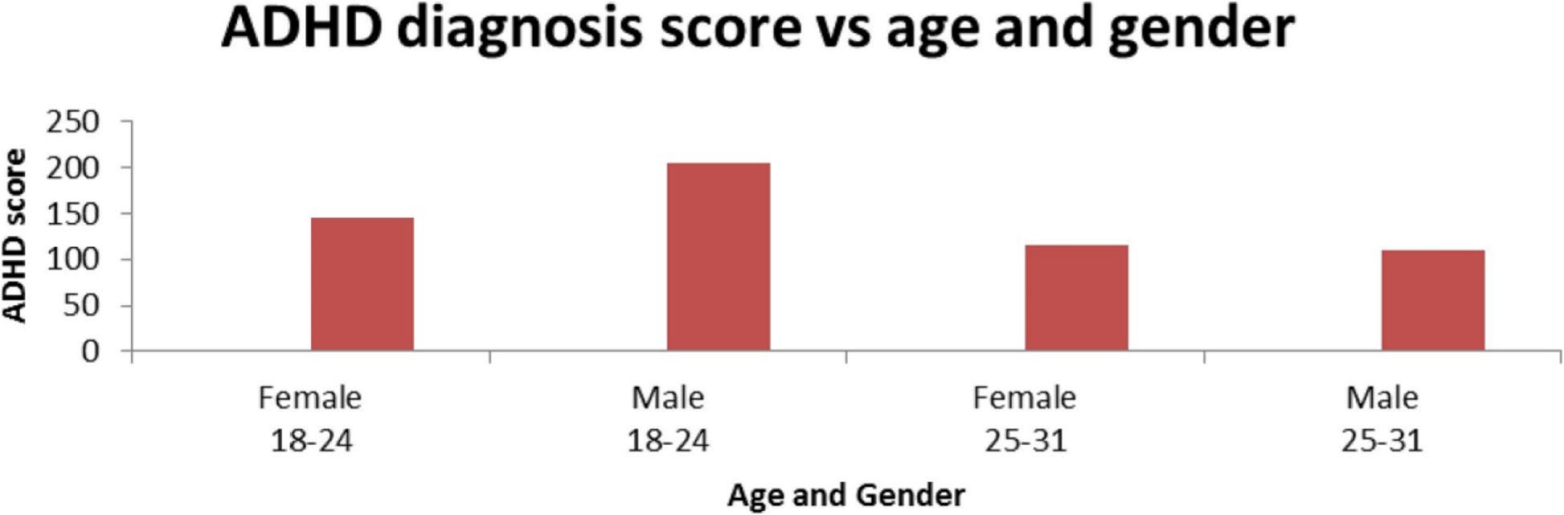
Distribution of Attention-deficit/hyperactivity disorder score in different age groups and gender

From Fig. 1, male participants' age ranged from 18–24 (37%) showed a higher ADHD score than the participants' age ranged from 25–31. Again, female participants with lower age were found with high ADHD scores. This showed a negative correlation with the age variation (*p* = 0.000).

**Fig. 2:**
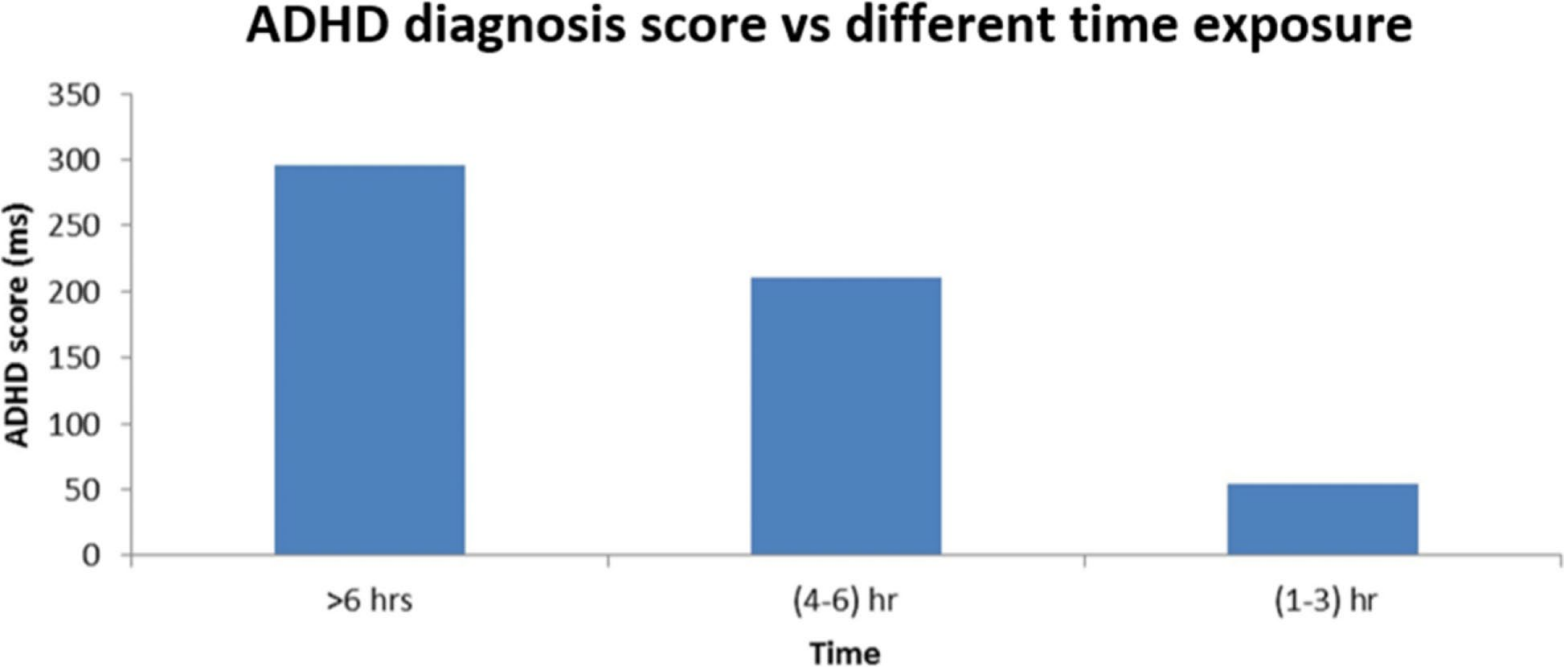
Distribution of Attention-deficit/hyperactivity disorder score in different time exposure of digital device

From Fig. 2, the ADHD score was found higher with the more time usage of the digital devices. ADHD score was found around 300 for greater than 6 h usage while found around 50 for 1–3 h usage.

In Table 2, the relationship between the Stroop effect and ADHD diagnosis score has been demonstrated. The Stroop effect has been measured by Stroop effect (ms) = (Incongruent RT -Congruent RT) [22].

**Table 2:** ADHD and Stroop effect correlation

		**ADHD score**	**Stroop effect**
ADHD score	Pearson correlation	1	0.498
	Sig. (2-tailed)		0.000
	N	197	197
Stroop effect	Pearson correlation	0.498	1
	Sig. (2-tailed)	0.000	
	N	197	197^a^

^a^Correlation is significant at the 0.01 level (2-tailed)

**Fig. 3:**
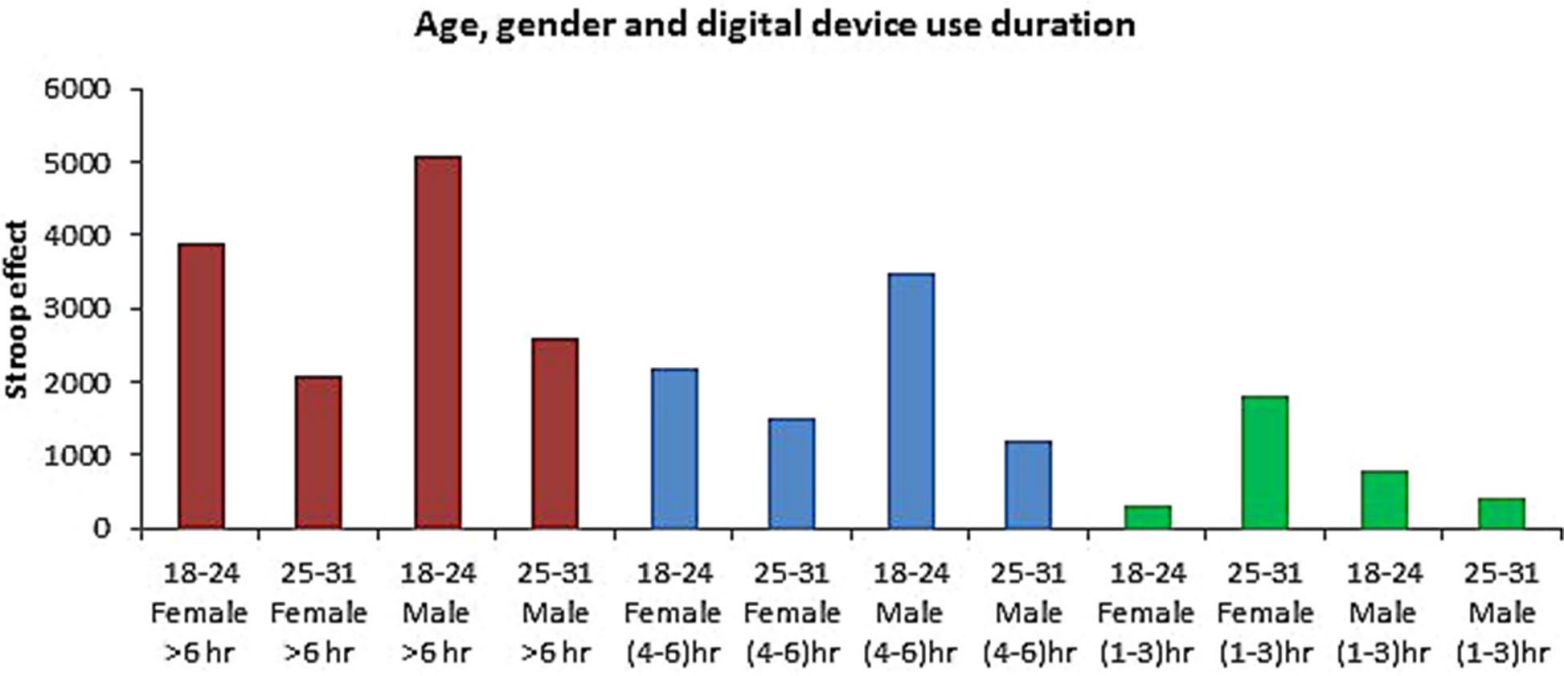
Changes of Stroop effect in different age, gender and digital device use durations

From the above graph (Fig. 3), it has been clearly stated that the Stroop effect is higher in the (18–24) aged male participants (48%) group for more than 6 h of digital device usage, and the Stroop effect value is lower for the (25–31) years aged male participants (10%) for the usage of 1–3 h.

From Table 3, Severe dry eye symptom was not found among the studied female population. Mild blurred vision was found higher among males (24%) and females (28%). Severe blurred vision was less prevalent among males (1%) and females (1%).

**Table 3:** CVS symptoms and the correlation with the gender

CVS symptoms	Gender				
	Male		Female		*p*-value
	N	Percentage	N	Percentage	
**Dry eyes**					
No effect	39	20%	52	26%	0.094
Mild	32	16%	22	11%	
Moderate	25	13%	24	12%	
Severe	3	2%	0	0%	
**Red and Irritated eyes**					
No effect	37	19%	39	20%	0.935
Mild	32	16%	28	14%	
Moderate	21	11%	21	11%	
Severe	9	5%	10	5%	
**Neck and Shoulder pain**					
No effect	27	14%	35	18%	0.591
Mild	33	17%	25	13%	
Moderate	30	15%	26	13%	
Severe	9	5%	12	6%	
Headache					
No effect	33	17%	38	19%	0.589
Mild	37	19%	31	16%	
Moderate	19	10%	21	11%	
Severe	10	4%	8	5%	
**Eye strain and feeling tired**					
No effect	20	10%	43	22%	0.018^*^
Mild	38	19%	22	11%	
Moderate	34	17%	24	12%	
Severe	7	4%	9	9%	
**Blurred vision**					
No effect	36	18%	33	17%	0.832^a^
Mild	47	24%	55	28%	
Moderate	15	8%	18	4%	
Severe	1	1%	2	1%	

^a^*p*-value less than 0.05 has been considered as statistically significant

^*^Correlation is significant in the spearman-Pearson correlation coefficient at the *p* = 0.05 level (2-tail)

From Table 4, Moderate eye strain and feeling tired were more prevalent (18%) as CVS symptoms among more than 6 h device users. Mild blurred vision was present among 4–6 h device users (24%) and 1–3 h device users (12%).

**Table 4:** CVS symptoms and correlation with the device used

CVS symptoms	Device use time						
	1–3 h		4–6 h		> 6 h		Correlation coefficient
	N	Percentage (%)	N	Percentage (%)	N	Percentage (%)	
**Dry eyes**							
No effect	40	20	21	15	30	11	0.263^**^
Mild	15	8	13	7	26	13	
Moderate	3	2	20	10	26	13	
Severe	1	1	1	1	1	1	
**Red and Irritated eyes**							
No effect	35	18	18	9	23	12	0.279^**^
Mild	18	9	14	7	28	14	
Moderate	4	2	16	8	22	11	
Severe	2	1	7	4	10	5	
**Neck and Shoulder pain**							
No effect	35	18	11	6	16	8	0.339**
Mild	13	7	19	10	26	13	
Moderate	10	5	18	9	28	14	
Severe	1	1	7	4	13	7	
**Headache**							
No effect	3	18	13	7	23	12	0.248**
Mild	13	7	25	13	30	15	
Moderate	9	5	12	6	19	10	
Severe	2	1	5	3	11	6	
**Eye strain and feeling tired**							
No effect	41	21	6	3	16	8	0.400**
Mild	10	5	26	13	24	12	
Moderate	6	3	17	9	35	18	
Severe	2	1	6	3	8	4	
**Blurred vision**							
No effect	32	16	15	8	22	11	0.238**^a^
Mild	31	12	34	24	47	16	
Moderate	3	2	8	4	12	6	
Severe	0	0	2	1	1	1	

^a,**^Correlation coefficient for the Pearson-spearman significance at the *p* = 0.01(2-tail)

## Discussion

With the advent of technologies, the prevalence of ADHD and CVS is increasing day by day. To the best of the authors' knowledge, no such study has been conducted previously on Bangladeshi people. From the study, it has been found that ADHD shows a strong correlation between gender and age (Fig. 1). Female participants aged 18–24 (24%) showed a significant relationship than those aged 25–31 (20%). A significant correlation (0.498**) has been found, exemplifying the positive linear relationship between ADHD score and the Stroop effect (Table 2). The gender and CVS symptoms correlation table, the eye-straining and feeling a tired symptom of CVS showed a significant (*p* = 0.018) (Table 3).

It has been elucidated that the time will be > 6 h (51%) in the ADHD score increase where the 1–3 h (12%) of the time increases. The evidence showed that ADHD symptoms are worsening for a selected group of students, which are the majority of heavy users, approximately 80% suggesting that a particular subset of teens may possess a greater vulnerability to ADHD symptoms [23]. Another study suggests that overuse of digital media is linked to increased attention problems in early adulthood and late adolescence/early adulthood [24]. Moreover, mid-adolescence is considered the time of neural plasticity when the brain circuit structured the attention and behavioral control, and the blue light exposure disrupts the development, and that is the reason for ADHD scoring in the 18–24 age group is higher than the 25- 31 group [25]. This study also found that the male group is the more vulnerable group for ADHD symptoms, and it is due to having an addiction to video games and more social media interaction than the female participants [26]. Figure 3 showed that the correlation with age, gender and digital device usage with the Stroop effect was established, where the 18–24 male (48%) showed the highest proximal of Stroop effect with the > 6 h digital device usage and the Stroop effect value is mostly lower for the 25–31 aged male participants (10%). As the color needs more attention than the word, the color interferes with the task [27]. The relatively faster processing affects slow processing and overall performance [10]. Moreover, in ADHD, the reduced visual attention and visual stimuli also reduced the overall performance and increased the reaction time for recognizing the color stimuli [22]. In sum, ADHD is the attention deficit disorder with or without hyperactivity where the Stroop task needs visual attention and fast processing time with no interference with the stimuli. Moreover, the neurocognition ability is hampered in the ADHD-like symptoms and heavy digital device usage, which is most needed in the Stroop task [28]. Table 3 showed that CVS significant positive correlation with the male gender, eye strain, and feeling tired (*p* = 0.018). The eye strain and eye fatigue pathophysiology are the repeated eye movement around the keyboard and the radiation from the digital device that hampered the eye cell and reduces the dopamine secretion from the brain which hampers our ocular nerve cell [3]. In this study, the eye-straining in the male group has been significant as the blue light has a detrimental effect on the eye, and males are more video-game addicted and Internet-addicted than females [25]. Blurred vision and double vision are other common symptoms of eye fatigue; continuous switching from light background to black background can disrupt the iris muscle and decrease blinking, which causes desiccation of the eye through an increased ocular surface area [25]. Moreover, lower resolution and high lightening also cause the red and irritated eye with a headache by decreasing dopamine level in the eye nerve cell and the eventual death of the eye nerve cell [4]. Because of the repetitive shifts and multitasking, which eventually impaired executive functioning and brain rest have been hampered too. Moreover, excessive technology use is associated with ADHD symptoms in adolescents and at any age. The light glaring and radiation decrease brain connectivity by decreasing the integrity of white matter pathways for reading and language [28]. The sleep cycle disruption also occurred due to the screen time increase and reduced functional connectivity, and increased risk for cognitive impairment at any age and the screen time correlation with eye-straining has been associated with the radiation, lightening and repetitive movement of eyes and switching to light to black and black to the light background [4].

There is minimal research-based data regarding ADHD in adults due to its challenging data collection, requiring skills and techniques that clinicians may not routinely use when assessing other disorders or age groups. That is why some underlying mechanism is still unknown [29]. Not all clinicians agree on conducting such assessments, and a substantial proportion reports a lack of confidence in their ability to reach accurate diagnostic judgments [29]. The strength of this study is that it has mainly focused on the young adults and adult groups' digital device use and ADHD and CVS symptoms prevalence, which is a very new attempt for addressing mental and physical related problems among this population group. It would contribute to develop interventions for preventing these abnormalities and might come handy to develop a specific guidelines for researching with the huge population and other factors also can be included. One of the major drawbacks in this study could be a less number of populations that have participated in this study and using the online platform for collecting data due to pandemic situations. Moreover, another limitation of  this study is that there has been a correlation with one environmental factor like digital media use without specifying the digital media content.

## Conclusions

ADHD and CVS are two emerging disorders in the modern world with the advancement of technology. The uses of digital devices have become essential nowadays due to the nature of work and structured professional systems. Unnecessary use of digital devices and specific protection with lifestyle modification may be helpful to reduce those disorders.

## Availability of data materials

Dataset used in this study will be available as per request (mailing to the corresponding author).

## Abbreviations

CVS: Computer vision syndrome
ADHD: Attention-deficit/hyperactivity disorder

## References

[1] Williams SG (2012) The ethics of internet research. Online J Nurs Informatics 16. 10.4135/9780857020055.n2

[2] Kesici A, Tunç NF (2018). Investigating the digital addiction level of the university students according to their purposes for using digital tools. Univers J Educ Res.

[3] Al Tawil L, Aldokhayel S, Zeitouni L (2020). Prevalence of self-reported computer vision syndrome symptoms and its associated factors among university students. Eur J Ophthalmol.

[4] Small GW, Lee J, Kaufman A (2020). Brain health consequences of digital technology use. Dialogues Clin Neurosci.

[5] Dos Santos Assef EC, Capovilla AGS, Capovilla FC (2007). Computerized stroop test to assess selective attention in children with attention deficit hyperactivity disorder. Span J Psychol.

[6] Ra CK, Cho J, Stone MD (2018). Association of digital media use with subsequent symptoms of attention-deficit/hyperactivity disorder among adolescents. JAMA - J Am Med Assoc.

[7] Smith M (2017). Hyperactive Around the World?. The History of ADHD in Global Perspective.

[8] Zhang J, Shuai L, Yu H (2020). Acute stress, behavioural symptoms and mood states among school-age children with attention-deficit/hyperactive disorder during the COVID-19 outbreak. Asian J Psychiatr.

[9] Merzon E, Manor I, Rotem A (2020). ADHD as a Risk Factor for Infection With Covid-19. J Atten Disord.

[10] Scarpina F, Tagini S (2017). The stroop color and word test. Front Psychol.

[11] Sheppard AL, Wolffsohn JS (2018). Digital eye strain: Prevalence, measurement and amelioration. BMJ Open Ophthalmol.

[12] Cardona G, García C, Serés C (2011). Blink rate, blink amplitude, and tear film integrity during dynamic visual display terminal tasks. Curr Eye Res.

[13] Dessie A, Adane F, Nega A (2018). Research Article.

[14] KEMP S, 2021 11 FEBRUARY Digital in Bangladesh: All the Statistics You Need in 2021 — DataReportal – Global Digital Insights. datareportal. Available from: https://datareportal.com/reports/digital-2021-bangladesh

[15] (2018) YES Centre (Youth Empowerment through Skills in Bangladesh) | YPSA [Internet]. ypsa 2018. Available from: http://ypsa.org/yes-centre-youth-empowerment-through-skills-in-bangladesh/

[16] Nur NS, Mullick MS1, Hossain A A (2018). ADHD is a Risk Factor of Road Crashes: A Purposive Study of Finding Relation between ADHD and Road Traffic Accident in Bangladesh. Int J Contemp Res Rev.

[17] Mowatt L, Gordon C, Santosh ABR, Jones T (2018) Computer vision syndrome and ergonomic practices among undergraduate university students. Int J Clin Pract. 72. 10.1111/ijcp.13035. 10.1111/ijcp.13035 28980750

[18] Kessler RC, Adler L, Ames M (2005). The World Health Organization adult ADHD self-report scale (ASRS): A short screening scale for use in the general population. Psychol Med.

[19] Kiatrungrit K, Putthisri S, Hongsanguansri S (2017). Validity and Reliability of Adult ADHD Self-Report Scale Thai Version (ASRS-V1.1 TH). Shanghai Arch Psychiatry.

[20] Green JG, DeYoung G, Wogan ME (2019). Evidence for the reliability and preliminary validity of the Adult ADHD Self-Report Scale v1.1 (ASRS v1.1) Screener in an adolescent community sample. Int J Methods Psychiatr Res.

[21] Song Y, Hakoda Y (2011). An asymmetric stroop/reverse-stroop interference phenomenon in ADHD. J Atten Disord.

[22] Algom D, Chajut E (2019) Reclaiming the Stroop effect back from control to input-driven attention and perception. Front Psychol. 10. 10.3389/fpsyg.2019.01683. 10.3389/fpsyg.2019.01683 PMC6688540 31428008

[23] Wiederhold BK (2019). Instagram: Becoming a worldwide problem?. Cyberpsychology, Behav Soc Netw.

[24] Yen CF, Ko CH, Yen JY (2009). Multi-dimensional discriminative factors for internet addiction among adolescents regarding gender and age. Psychiatry Clin Neurosci.

[25] Lissak G (2018). Adverse physiological and psychological effects of screen time on children and adolescents: Literature review and case study. Environ Res.

[26] Banaschewski T, Becker K, Döpfner M (2017). Attention-deficit/hyperactivity disorder-a current overview. Dtsch Arztebl Int.

[27] Cothran DL, Larsen R (2008). Comparison of inhibition in two timed reaction tasks: The color and emotion stroop tasks. J Psychol Interdiscip Appl.

[28] Blehm C, Vishnu S, Khattak A (2005). Computer vision syndrome: A review. Surv Ophthalmol.

[29] Lovett BJ, Harrison AG (2021). Assessing adult ADHD: New research and perspectives. J Clin Exp Neuropsychol.

